# Tyrosine Dephosphorylation of ASC Modulates the Activation of the NLRP3 and AIM2 Inflammasomes

**DOI:** 10.3389/fimmu.2019.01556

**Published:** 2019-07-05

**Authors:** Bezaleel Mambwe, Kurt Neo, Hanif Javanmard Khameneh, Keith Weng Kit Leong, Mariasilvia Colantuoni, Maurizio Vacca, Richmond Muimo, Alessandra Mortellaro

**Affiliations:** ^1^Singapore Immunology Network (SIgN), Agency for Science, Technology and Research (A^*^STAR), Singapore, Singapore; ^2^Department of Infection, Immunity and Cardiovascular Diseases, The University of Sheffield, Sheffield, United Kingdom; ^3^San Raffaele Telethon Institute for Gene Therapy (SR-Tiget), IRCCS San Raffaele Scientific Institute, Milan, Italy; ^4^International PhD Program in Molecular Medicine, Vita-Salute San Raffaele University, Milan, Italy

**Keywords:** inflammasome activation, NLRP3, ASC, tyrosine dephosphorylation, tyrosine phosphorylation

## Abstract

The inflammasome is an intracellular multi-protein complex that orchestrates the release of the pro-inflammatory cytokines IL-1β and IL-18, and a form of cell death known as pyroptosis. Tyrosine phosphorylation of the inflammasome sensors NLRP3, AIM2, NLRC4, and the adaptor protein, apoptosis-associated speck-like protein (ASC) has previously been demonstrated to be essential in the regulation of the inflammasome. By using the pharmacological protein tyrosine phosphatase (PTPase) inhibitor, phenylarsine oxide (PAO), we have demonstrated that tyrosine dephosphorylation is an essential step for the activation of the NLRP3 and AIM2 inflammasomes in human and murine macrophages. We have also shown that PTPase activity is required for ASC nucleation leading to caspase-1 activation, IL-1β, and IL-18 processing and release, and cell death. Furthermore, by site-directed mutagenesis of ASC tyrosine residues, we have identified the phosphorylation of tyrosine Y60 and Y137 of ASC as critical for inflammasome assembly and function. Therefore, we report that ASC tyrosine dephosphorylation and phosphorylation are crucial events for inflammasome activation.

## Introduction

The inflammasome is a multiprotein complex comprising of a sensor receptor protein, an adaptor protein apoptosis associated speck-like protein (ASC) and the cysteine protease caspase-1 that upon activation leads to the maturation and secretion of IL-1β and IL-18 cytokines, and a form of inflammatory cell death known as pyroptosis (1). Inflammasome complexes are named according to the sensor protein engaged by the danger signal. For instance, the NLRP3 inflammasome is activated by K^+^ efflux caused by a plethora of agonists, while the AIM2 inflammasome is formed in the presence of viral and bacterial double-stranded DNA (2, 3).

The NLRP3 inflammasome has been widely studied and its constitutive activation is associated with autoinflammatory diseases caused by gain-of-function mutations in the NLRP3 gene, known as Cryopyrin associated periodic syndromes (CAPS) (4–7). Overt NLRP3 inflammasome activation has been shown to be associated with detrimental inflammation in various cancers, Alzheimer's disease and obesity (5–7). With such a widespread impact on disease mechanisms, understanding the regulation of the NLRP3 inflammasome complex is vital in the development of therapies to alleviate the inflammatory symptoms caused by NLRP3 inflammasome hyper-activation.

Protein phosphorylation of specific amino acid residues has been shown to direct inflammasome activation at mainly the sensor and adaptor protein level (8). It was reported that NLRC4 is phosphorylated at serine 533 by the kinase Protein Kinase C Delta (PRKCD/PKCδ) following detection of Type III secretion systems (T3SS) and bacterial flagellin leading to the formation of the NLRC4 inflammasome (9). Furthermore, it has been shown that Bruton's tyrosine kinase (BTK), anaplastic lymphoma kinase (ALK), and Protein Kinase D (PKD) can phosphorylate NLRP3. NLRP3 is also dephosphorylated by protein tyrosine phosphatase non-receptor 22 (PTPN22) at Y861 and protein phosphatase 2A (PP2A) at S5 leading to its activation (10–13). Similarly, ASC is also subjected to phosphoryl modifications by kinases, such as spleen tyrosine kinase (Syk) and c-Jun N-terminal kinase (JNK) (14), which act on the kinase Pyk2 to phosphorylate murine ASC at tyrosine 144 (corresponding to human Y146) leading to ASC nucleation in inflammasome activation (15). In addition, Iκβ kinase alpha (IKKα) activity has been shown to negatively regulate the NLRP3, NLRC4, and AIM2 inflammasomes by interacting with and sequestering ASC to the nucleus. Following inflammasome agonist detection, the IKKα-ASC interaction is inhibited by PP2A thus leading to cytosolic translocation of ASC, which is then able to interact with the receptor proteins (16).

However, the requirement of tyrosine dephosphorylation of ASC for inflammasome activation needs to be investigated. We thus assessed the role of protein tyrosine phosphatases (PTPases) in regulating NLRP3 and AIM2 inflammasomes using phenylarsine oxide (PAO), a membrane-permeable PTPase inhibitor that binds to vicinal thiols in the target protein (17). We found that PAO mediated NLRP3/AIM2 inflammasome inhibition was due, in part, to the inhibition of ASC tyrosine dephosphorylation. Furthermore, by site-directed mutagenesis, we identified the tyrosine ASC residues that are required for normal inflammasome function. The understanding of the tyrosine phosphorylation mechanisms underpinning the role of ASC would be the basis on which to explore tyrosine phosphorylation targeting therapeutic interventions.

## Methods

### Mice

C57BL/6J mice were obtained from the Biological Resource Center (BRC), Agency for Science, Technology and Research (A^*^STAR), Singapore, and used to isolate bone marrow progenitors from femurs and tibia for the differentiation *in vitro* of mouse macrophages. Mice were maintained under specific pathogen-free conditions. All experimental procedures were approved by the Institutional Animal Care and Use Committee (IACUC) of the BRC (A^*^STAR, protocol #0161113) in compliance with their Guidelines for Animal Experiments.

### Cell Culture

Mouse macrophages (BMDMs) were *in vitro* differentiated from bone marrow progenitors isolated from femurs and tibia of C57BL/6J mice cultured for 8 days in complete Iscove's Modified Dulbecco's Medium (IMDM) (10% fetal bovine serum, 1% penicillin/streptomycin and glutamine) supplemented by 10% L929-conditioned medium containing M-CSF. Human THP-1 monocytic cells were differentiated into macrophage-like cells with phorbol 12-myristate13-acetate (PMA, 100 nM, Sigma Aldrich) in complete RPMI-1640 (Gibco) overnight (treatment phase), then followed by 1-day in PMA-free media (resting phase) and then used for experiments on day 3. HEK293 cells were cultured in complete Dulbecco's modified Eagle's medium (DMEM) prior to and during transfections.

### Inflammasome Activation and Phosphatase Inhibition

THP-1 macrophages and BMDMs were primed with LPS from *E.coli* (1 μg/ml, Enzo Life Sciences) for 3 h. To activate the NLRP3 inflammasome, nigericin (10 μM), or monosodium urate crystals (MSU; 200 μg/ml) was added to the cells for 1 and 6 h, respectively. The AIM2 inflammasome was activated by transfection with poly(dA:dT) (1 μg/ml with 1 μl of Lipofectamine 2000, Invitrogen) for 6 h after a 3-h LPS treatment. To inhibit protein tyrosine phosphatases, phenylarsine oxide (PAO, Sigma-Aldrich) was added to the culture the last 30 min of the LPS treatment, and then the inflammasome activator was added.

### Cytokine Measurements

IL-1β, TNF (R&D Systems) and IL-18 (MBL) were measured in the cell supernatants by ELISA according to the manufacturer's instructions.

### Cell Death Assay

Lactate dehydrogenase (LDH) levels in cell-free culture supernatants were assessed using the CytoTox 96® Non-Radioactive Cytotoxicity kit (Promega) according to manufacturer's instructions.

### Acetone Protein Precipitation

To assess the secretion of processed forms of IL-1β and caspase-1, the hour-long nigericin treatment (±PAO following signal 1) was carried out in serum-free media. Equal amounts of the serum-free media from each condition were collected and individually precipitated for 1 h at −20°C in acetone at a 4X volume of the sample. The precipitated proteins were resuspended in Laemmli buffer and subjected to western blot analysis.

### Disuccinimidyl Suberate (DSS)-Mediated Cross-Linking

Following cell treatment, disuccinimidyl suberate (DSS)-mediated protein crosslinking was carried out to assess ASC oligomerization, as previously described (18).

### Western Blot Analysis

Thirty to fifty micrograms (30–50 μg) of lysates (or acetone precipitated/ DSS-oligomerized protein samples) were incubated in Laemmli sample buffer for 5 min at 100°C. The denatured protein samples were separated by SDS-PAGE followed by protein transfer to PVDF membrane (Millipore). The membranes were then blocked with 5% milk (or 5% BSA for phosphoprotein analysis) in TBS-Tween-20 at room temperature for 1 h. The blots were then incubated overnight with the following primary antibodies: anti-ASC (B-3 1:1,000; N-15-R 1:500), anti-pTyr (pY99, 1:1,000), anti-IL-1β (H153, 1:1,000), anti-caspase-1 (M-20, 1:500), and anti-β-actin (C-4, 1:1,000) antibodies (all from Santa Cruz); anti-GAPDH antibody (Millipore-MAB374, 1:1,000) from Millipore. Following overnight incubation, the membranes were washed and then incubated in the corresponding Horseradish Peroxidase (HRP) conjugated secondary antibody (Dako, Millipore) for 1 h at room temperature, followed by a multi-wash step. Membranes were developed with Supersignal™ West Pico chemiluminescence detection (Pierce) solution and visualized by a chemiluminescence imager (Bio-Rad).

### Confocal Microscopy

THP-1 macrophages were cultured in chamber microscope slides at a density of 150,000 per chamber. Following inflammasome activation, cells were fixed with 2% paraformaldehyde at room temperature for 10–15 min, and permeabilized in permeabilization buffer (0.1% saponin, 0.2% gelatin, 5 mg/ml BSA, and 0.02% sodium azide in PBS), followed by three 10-min washes with PBS. Cells were incubated in blocking solution (0.01% saponin, 0.2% gelatin, and 5 mg/ml BSA in PBS) for 45–60 min at room temperature under agitation, followed by ASC labeling with the anti-ASC (SCBT N-15-R, 1:50) antibody for 1 h at room temperature or overnight at 4°C. Cells were washed 3 times for 10 min each with PBS, and then the secondary anti-Rabbit Alexa Fluor-488-conjugated antibody (Life Technologies, 1:1,000) was added for 1 h at room temperature on the dark. Nuclear staining was carried out by adding 4',6-diamidino-2-phenylindole (DAPI) on the third wash. The images were taken with an Olympus confocal microscopy system (FV-1000 inverted Olympus IX81microscope, magnification of 100X, 2X digital zoom) and speck count was done using ImageJ software. At least 7 fields of ≥100 cells were blindly selected and counted per condition. The nuclei to ASC speck ratio was calculated and multiplied by 100 to produce a percentage for comparison across conditions. Statistical analysis was carried out with the two-tailed paired *t*-test with Gaussian distribution (parametric test) with the GraphPad Prism software.

### Protein Immunoprecipitation

Lysates (0.5–1.0 mg) obtained with complete RIPA buffer containing 1 mM sodium orthovanadate and protease inhibitor cocktail were initially pre-cleared with protein G sepharose beads for 1 h at 4°C. The preclear mix was centrifuged at maximum speed for 30 s at 4°C and the supernatant collected, and the primary anti-pTyr-pY99 (SCBT, cat. sc-7020), anti-ASC (Adipogen, cat. AL177), and IgG from mouse serum (Sigma Aldrich, cat. I5381-1MG) antibodies were added. The antibody-supernatant mix was incubated for 2 h or overnight at 4°C with agitation. Protein G coated beads were added to the supernatant-antibody complex and incubated overnight at 4°C. Then the mix was centrifuged, washed twice with complete RIPA buffer, and incubated with 30–60 μl of Laemmli buffer for 5 min at 100°C prior to protein separation on SDS-PAGE.

### Generation of ASC Mutant Plasmids

Tyrosine to phenylalanine (Y>F) ASC mutants of the pEF6-ASC-GFP plasmid were generated using the Q5 site-directed mutagenesis kit (New England Biolabs) according to the manufacturer's instructions. The mutated plasmids were transformed into DH5α chemically competent *E. coli* and then isolated by mini- or midi-preps (Qiagen). The isolated plasmids were then sequenced to validate the mutations.

### Transfection of HEK293 Cells

HEK293 cells were seeded into 24 well plates at a cell density of 200,000 cells per well and incubated overnight. Cells were then transfected with pCR3-NLRP3-FLAG (100 ng), pEF6-ASC-GFP and validated mutants (50 ng), pCI-pro-caspase-1 (30 ng, Addgene #41552), pcDNA3-pro-IL-1β (100 ng), and empty pCR3.1 vector (620 ng) with Lipofectamine 2000 (Life Technologies) for 24 h. Media was changed and cells incubated for an additional 24 h. Forty-eight hours later, the media was collected for ELISA analysis and the lysate prepared for western blot analysis.

### Real-Time RT-PCR

Total RNA was isolated using Trizol® (Invitrogen) followed by PureLink™ RNA Mini Kit (ThermoFisher). Genomic DNA was removed using a PureLink™ DNase Set (ThermoFisher). RNA retrotranscription was performed using a High Capacity cDNA Reverse Transcription Kit (Applied Biosystems). Semi-quantitative RT-PCR was performed in triplicates using QuantiFast® SYBR® Green PCR kit (Qiagen) and the following primers: IL-1β forward 5′-CCAGTGAAATGATGGCTTATTAC-3′, reverse 5′-CTGTAGTGGTGGTCGGAGATT-3′; IL-8 forward 5′-CATCTCACTGTGTGTAAACATGAC-3′, reverse 5′-CCTTGGCAAAACTGCACCTTCAC-3′; GAPDH forward 5′-CCACATCGCTCAGACACCAT-3′, reverse 5′-GGCAACAATATCCACTTTACCAGAGT-3′. Amplification was performed on a ViiA 7 Real-Time PCR System. The relative expression level of each gene was evaluated by the 2-DDCt method. The difference between Ct of the target gene and Ct of the housekeeping gene GAPDH was normalized to the difference between the Ct of the same genes in the untreated condition used as calibrator.

### Statistical Analysis

Statistical analysis was carried out using the GraphPad Prism Software. All ELISA data were analyzed by ordinary One-Way ANOVA and ASC speck count data was analyzed by two-tailed paired *t*-test with Gaussian distribution (parametric test).

## Results

### PAO Inhibits NLRP3 Inflammasome Activation

**Figure 1:**
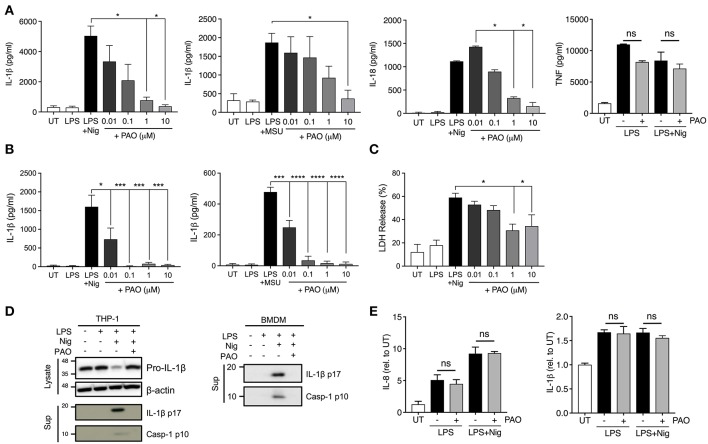
PAO inhibits NLRP3 inflammasome activation. THP-1 macrophages **(A,C–E)** and BMDMs **(B,D)** were primed with LPS (1 μg/ml) for 3 h followed by treatment with PAO (0.01–10 μM) or vehicle for 30 min. Nigericin (10 μM) or MSU crystals (200 μg/ml) were then added for 1 and 6 h, respectively, to activate the NLRP3 inflammasome. IL-1β, IL-18, and TNF release was assessed in cell-free supernatants of THP-1 macrophages **(A)** and BMDMs **(B)** by ELISA. **(C)** LDH measurement was carried out on THP-1 macrophage supernatants to evaluate cell death. **(D)** Pro-IL-1β level and processing of IL-1β and caspase-1 were assessed by immunoblot in THP-1 macrophages and BMDMs. **(E)** Pro-IL-1β and IL-8 transcript levels were measured in THP-1 macrophages by real-time RT-PCR. Data represent the means ± standard error of three independent experiments. Statistical analysis was carried out by comparing each of the inhibitor treatment conditions to the LPS+Nig or LPS+MSU condition using ordinary one-way ANOVA with the Dunnett test. *p ≤ 0.05; ***p ≤ 0.001; ****p ≤ 0.0001; ns, *p* > 0.05.

The vital role that tyrosine phosphorylation plays in the regulation of protein function is indisputable. To assess the role of tyrosine phosphorylation in the formation and function of the inflammasome complex we used PAO, a broad-spectrum PTPase inhibitor (19). PAO has been identified as an inhibitor of protein tyrosine phosphatases by binding to the catalytic site of the target protein tyrosine phosphatase (17, 20). Human THP-1 macrophages and mouse bone marrow-derived macrophages (BMDMs) were primed with LPS for 3 h followed by addition of increasing concentrations of PAO (0.01–10 μM or vehicle) for 30 min. Then, cells were stimulated with nigericin (10 μM) or MSU (200 μg/ml) to activate the NLRP3 inflammasome. PAO inhibited IL-1β release by THP-1 macrophages (Figure 1A) and BMDMs (Figure 1B) in a dose-dependent fashion. Similarly, nigericin-induced IL-18, but not TNF, release by THP-1 macrophages was also reduced (Figure 1A). Moreover, we found that PAO also inhibited cell death of THP-1 macrophages following nigericin stimulation (Figure 1C). The PAO-mediated inhibition of nigericin-induced cell death occurred at the same concentration as the inhibition of IL-1β release (Figure 1A).

To further understand whether PAO interferes with the priming or activation step of NLRP3 inflammasome activation we examined the pro-caspase-1 and pro-IL-1β expression, processing and release by western blot. The cleaved and secreted forms of both caspase-1 (p10) and IL-1β (p17) were undetectable in the supernatant of nigericin-activated THP-1 cells and BMDMs in the presence of PAO (Figure 1D, Supplementary Figures 1A,B). PAO treatment did not alter pro-IL-1β protein expression (Figure 1D, Supplementary Figure 1C). Also, mRNA levels of pro-IL-1β and IL-8 (non-inflammasome cytokine) were unchanged by PAO compared to vehicle control (Figure 1E). These data indicate that PAO inhibits NLRP3 inflammasome activation but not the priming step.

### PAO Inhibits ASC Oligomerization and Speck Formation

**Figure 2:**
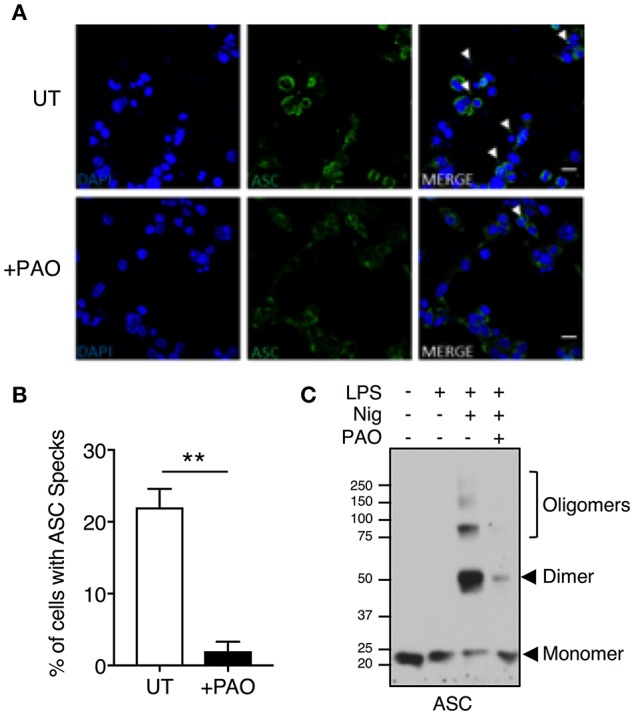
Inhibition of tyrosine dephosphorylation hinders ASC aggregation. THP-1 macrophages were primed and treated with PAO (1 μM) for 30 min. Nigericin (10 μM) was then added for 30–45 min. **(A,B)** Cells were fixed (2% paraformaldehyde) and immuno-stained with an anti-ASC antibody followed by anti-rabbit Alexa Fluor-488-conjugated secondary antibody then visualized by confocal microscopy (Magnification 100X, scale bar 20 μm). The number of THP-1 macrophages containing an ASC speck per nucleus (DAPI stained) was counted and calculated as a percentage. All data represent the means ± standard error of a representative experiment out of three. Two-tailed paired *t*-test was carried out with Gaussian distribution assumed (parametric test) **p ≤ 0.01. **(C)** DSS-mediated crosslinking of ASC monomers was carried out and analyzed by immunoblot. Representative image of *n* = 3.

ASC speck formation is a hallmark of inflammasome activation. To investigate whether the effect of PAO is upstream of caspase activation, we analyzed speck formation in LPS-primed THP-1 macrophages stimulated with nigericin in the presence or absence of PAO treatment. ASC specks were readily visible in nigericin-stimulated THP-1 macrophages and significantly reduced in the presence of PAO, as assessed by confocal microscopy (Figures 2A,B).

To further confirm the PAO-mediated disruption of ASC speck formation, an ASC oligomerization assay was carried out by DSS-mediated crosslinking. Nigericin treatment led to ASC oligomerization with the protein mostly existing as a dimer, whereas ASC oligomerization was attenuated in the presence of PAO (Figure 2C). These data indicate that PAO acts at the nucleation step of the ASC filament formation upstream of caspase-1.

### PAO Inhibits the AIM2 Inflammasome

**Figure 3:**
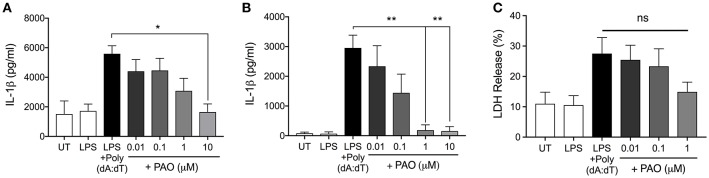
PAO inhibits the AIM2 inflammasome. LPS-primed THP-1 macrophages and BMDMs were transfected with poly(dA:dT) (1 μg/μl) for 6 h in the presence or absence of increasing doses of PAO (0.01–1 μM) or with the vehicle. ELISA was carried out to measure IL-1β release from THP-1 macrophages **(A)** and BMDMs **(B)**. **(C)** LDH measurement was carried out on THP-1 macrophages supernatants to assess cell death. All data represent the means ± standard error of three experiments. Statistical analysis was carried out by comparing each of the inhibitor treatment conditions to the LPS+Poly(dA:dT) condition using ordinary one-way ANOVA with the Dunnett test. *p ≤ 0.05; **p ≤ 0.01; ns, *p* > 0.05.

We next asked whether PAO could block other inflammasomes. The AIM2 inflammasome is formed following the detection of bacterial and viral double-stranded DNA (3). Experimentally, synthetic double-stranded DNA, poly(deoxyadenylic-deoxythymidylicpolyic) [poly(dA:dT)] serves as an activator of the AIM2 inflammasome (3). Following LPS priming, THP-1 macrophages and BMDMs were treated with increasing concentrations of PAO or vehicle followed by cytosolic transfection of poly(dA:dT) for 6 h. PAO inhibited poly(dA:dT)-induced IL-1β release by THP-1 macrophages and BMDMs in a dose-dependent manner, similarly to nigericin and MSU-mediated NLRP3 inflammasome activation (Figures 3A,B). Poly(dA:dT)-mediated cell death of THP-1 macrophages assessed as lactate dehydrogenase (LDH) release was also attenuated by PAO treatment (though not statistically significant) (Figure 3C). These data indicate that PTPase activity is important for the activation of NLRP3 and the AIM2 inflammasomes.

### Phosphorylation Status of Tyrosines Y60 and Y137 Regulates ASC Function

**Figure 4:**
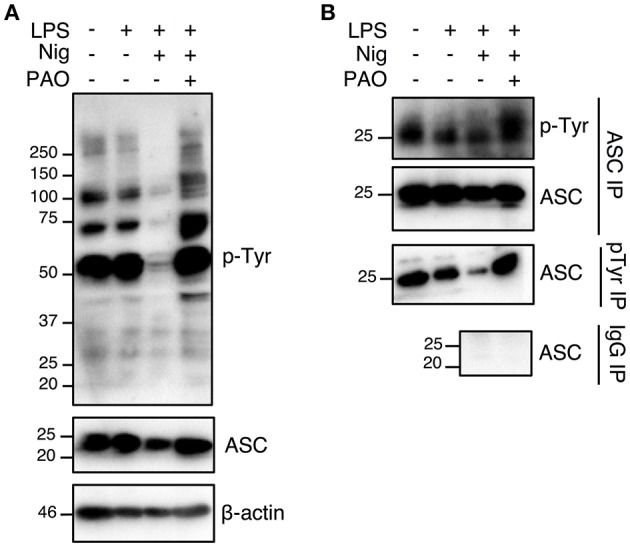
The nigericin-induced global and ASC tyrosine dephosphorylation is inhibited by PAO. THP-1 macrophages were primed followed by treatment with PAO (1 μM) for 30 min. Nigericin (10 μM) was then added for 1 h. **(A)** Immunoblot analysis was carried out on total cell lysates for tyrosine phosphorylation and ASC using anti-pTyr (pY99) and anti-ASC antibodies, respectively. β-actin was used as a loading control. **(B)** Immunoprecipitations of ASC and phosphotyrosine proteins were carried out followed by immunoblots for pTyr and ASC, respectively. Immunoprecipitation using beads coated by mouse IgG was performed as negative control. Data is representative experiment out of three.

Nigericin treatment induced a general protein tyrosine dephosphorylation, which was inhibited by the addition of PAO, indicating that PAO effectively inhibited protein tyrosine phosphatase activity (Figure 4A, Supplementary Figures 1D,E). We next determined the tyrosine phosphorylation status of ASC in THP-1 macrophages by immunoprecipitation analysis. We found that ASC was in a state of tyrosine phosphorylation at the steady state in THP-1 macrophages, and stimulation by LPS+nigericin induced tyrosine dephosphorylation of ASC (Figure 4B, Supplementary Figures 1D,E). ASC tyrosine dephosphorylation was inhibited by the addition of PAO (Figure 4B, Supplementary Figures 1D,E). Furthermore, our negative IgG control revealed that there is no non-specific IgG binding to ASC in the immunoprecipitation (Figure 4B, Supplementary Figure 1E). This also shows that there is no non-specific binding of the IgG to pTyr since ASC, a protein phosphorylated at tyrosine, is not immunoprecipitated. Therefore, our data suggests that ASC tyrosine dephosphorylation is a necessary step in NLRP3 inflammasome complex formation and function.

**Figure 5:**
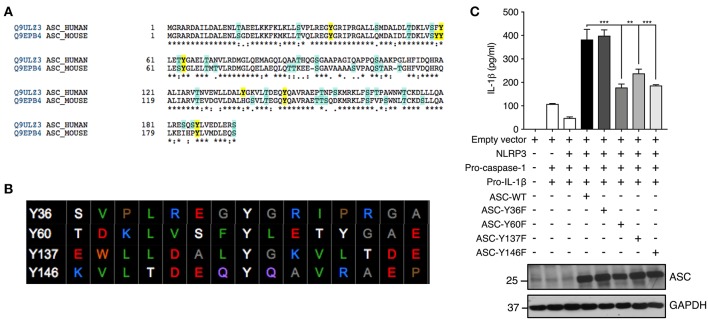
Residues Y60 and Y137 are required for ASC function. **(A,B)** Tyrosine residue selection criteria were based on conservation of the residues between mouse and human proteins and predicted by the online predication algorithm phosphoNET to undergo phosphorylative post-translational modifications. **(C)** Site-directed mutagenesis of Y36, Y60, Y137, and Y146 to phenylalanine was carried out and the NLRP3 inflammasome was reconstituted in HEK293T cells by transfection of NLRP3, pro-casp-1, pro-IL-1β, and validated ASC variants. IL-1β ELISA was carried out to measure the function of the reconstituted NLRP3 inflammasome. Representative image of immunoblot analysis of ASC to determine equal transfection (*n* = 3). GAPDH was used as loading control for the immunoblot. Statistical analysis was carried out by comparing each of the mutant transfection conditions to the ASC-WT condition using ordinary one-way ANOVA with the Dunnett test. **p ≤ 0.01, ***p ≤ 0.001.

We further investigated ASC tyrosine phosphorylation by site-directed mutagenesis of the specific tyrosine residues Y36, Y60, Y137, and Y146 to phenylalanine, a non-phosphorylatable tyrosine mimic. This Y>F mutation maintains the protein structure but prevents phosphorylation at the mutated site because phenylalanine lacks a hydroxyl group required for the addition of a phosphoryl group. The tyrosine residues of ASC protein were selected with the following criteria: conservation between mouse and human and predicted to be phosphorylated by an online phosphorylation site prediction algorithm (PhosphoNET: http://www.phosphonet.ca) (Figures 5A,B). We utilized Y146 as our positive control as it has already been shown to be required for ASC function (14, 15, 21). Following mutagenesis of tyrosine to phenylalanine, the wild type and mutant ASC plasmids were co-transfected into HEK293T cells along with plasmids encoding human NLRP3, pro-caspase-1 and pro-IL-1β for 48 h. We found that similarly to Y146, Y60, and Y137 ASC mutants generated exhibited a diminished inflammasome activity measured by IL-1β release, compared to the wild type ASC, indicating that Y60 and Y137 residues require tyrosine phosphorylation for normal ASC function (Figure 5C, Supplementary Figure 1F). Conversely, the Y36F ASC mutant does not exhibit reduced functionality (Figure 5C, Supplementary Figure 1F), indicating that Y36 phosphorylation is not required.

## Discussion

In the present report, using a pharmacological inhibitor and site-directed mutagenesis we have shown that tyrosine dephosphorylation of the inflammasome adaptor ASC by PTPases is a crucial step for the activation of NLRP3 and AIM2 inflammasomes in human and murine macrophages. Furthermore, we identified the phosphorylation of tyrosine Y60 and Y137 of ASC as a critical event for inflammasome assembly and function, including ASC nucleation, caspase-1 activation, IL-1β, and IL-18 processing and release, and cell death.

This is the first time that PAO has been shown to elicit an effect on nigericin-induced NLRP3 inflammasome functionality. The IL-1β mechanism of release has been attributed to the loss of cell membrane integrity as a consequence of necrotic or pyroptotic cell death (22, 23). We have shown that inflammasome-induced cell death is attenuated in the presence of PAO and this might explain the lack of IL-1β release (due to inhibition of membrane permeability). Indeed, we showed that pro-IL-1β expression level is unaltered while the processing is disrupted. Alternatively, pro-IL-1β processing could be perturbed due to PAO-mediated attenuation of caspase-1 activation, according to Takahashi et al. (24). Since caspase-1 activation is dependent on ASC aggregation, analysis of ASC nucleation and speck formation showed that PAO inhibits ASC oligomerization and formation of ASC specks. This indicates that the PAO-elicited inhibitory effect is upstream of caspase-1 processing.

The AIM2 inflammasome, activated by intracellular double stranded DNA, is formed with the same components as the NLRP3 inflammasome with exception of the sensor protein. In our study, treatment with PAO similarly resulted in attenuation of IL-1β release and cell death in both human and murine macrophages. This demonstrates that PAO acts upon a pathway that is common in the regulation of these two inflammasome complexes.

To further dissect the mechanism involved in PAO-mediated inflammasome inhibition, we showed that PAO inhibited nigericin induced global dephosphorylation of tyrosine residues, implicating the inhibition of some PTPase activity in inflammasome suppression and suggesting that tyrosine dephosphorylation is critical for the activation of the inflammasome. In contrast to PAO, sodium orthovanadate (OVN), a competitive inhibitor that competes with phosphate for the catalytic site of PTPases (25), augmented IL-1β release (26). However, the long treatment durations employed in this study suggests the observed effects more likely resulted from gene transcription rather than a direct effect of OVN on inflammasome activation. In another study, pre-treatment with OVN (1 mM) for 1-h prior to ATP treatment augmented ATP-induced IL-1β release in murine cells. However, at lower concentrations, OVN had no impact. Interestingly, Hoyt and colleagues also showed that while ATP induced global tyrosine dephosphorylation and IL-1β release, ethanol induced further global dephosphorylation but blocked IL-1β release. Furthermore, OVN reversed the ethanol-mediated inhibition of IL-1β release in a concentration dependent manner, suggesting that these processes likely occur independently. In this regard, Hoyt et al. also showed that both the OVN and ATP-induced ASC phosphorylation at Y144 was abrogated by ethanol. However, the OVN-induced p-ASC at Y144 did not elicit IL-1β release (21). Put together, the differences observed between PAO and OVN could be due to differences in protein targets and mechanisms of action. Whilst there are common targets of OVN and PAO, there could be PAO-sensitive non-PTPase targets that are involved in inflammasome activation. For instance, the non-PTPase NADPH oxidase 2 (NOX2) is inhibited by PAO (27–29) and has been shown to regulate oxidative stress-induced NLRP3 inflammasome activation in murine neural tissue (30). Furthermore, PAO can inhibit non-tyrosine phosphatases, such as the calcium and calmodulin-dependent serine/threonine phosphatase calcineurin in the bovine brain (31). In calcineurin transgenic mice, where calcineurin is constitutively active, the NLRP3 inflammasome is activated implying that calcineurin plays an activatory role in NLRP3 inflammasome in myocardial tissue (32). Another target for PAO is RhoA GTPase (19), however RhoA has only been reported to be involved in the pyrin inflammasome (33). Taken together, although NOX2, calcineurin and RhoA GTPase are PAO-sensitive, inhibition by PAO does not account for the observed inhibition of both NLRP3 and AIM2 inflammasomes in our investigation where human and murine macrophages with concentrations of 1 μM and 0.1 μM (respectively) have significantly reduced NLRP3 inflammasome function. In the case of calcineurin, it would be interesting to assess whether calcineurin is involved in nigericin-mediated NLRP3 inflammasome function. Since we observed that ASC tyrosine dephosphorylation is inhibited by PAO, we hypothesize that PAO acts on a pathway that regulates tyrosine dephosphorylation of ASC upon inflammasome activation, thereby implicating one or more PTPases that have a higher affinity for PAO compared to OVN.

ASC phosphorylation at Y146 (corresponding to Y144 in mouse) by Pyk2 has been shown to be necessary for ASC function (14, 15). Our immunoprecipitation data suggests that ASC is perpetually phosphorylated at tyrosine and dephosphorylated upon stimulation with nigericin to form the NLRP3 inflammasome. The phosphorylative regulation of NLRP3 has already been demonstrated, where an NLRP3 agonist enlists tyrosine kinases (BTK, ALK) (10) and tyrosine phosphatase PTPN22 (11) to phosphorylate and dephosphorylate NLRP3 resulting in inflammasome activation. Therefore, since we have now demonstrated that dephosphorylation of ASC also takes place, we hypothesize that ASC is regulated by tyrosine kinases (*e.g.*, Pyk2) and a yet to be identified protein tyrosine phosphatase upon inflammasome activation leading to ASC aggregation and speck formation.

The Y144 residue of ASC in mouse is conserved in human (Y146), which indicates that this residue is required for normal function. It has previously been demonstrated that mutation of the Y144/6 residue resulted in the inability of ASC to form specks (14, 15), therefore, we sought to identify and characterize putative ASC tyrosine residues. The Y36, Y60 and Y137 residues were identified and mutated to phenylalanine (F) which mimics constitutive dephosphorylation and prevents phosphorylation without much disruption to overall protein structure. We showed that Y60 and Y137 residues require phosphorylation for optimal ASC function but not Y36. The Y36F mutant functioned as the wild type which suggests two possibilities; phosphorylation at this residue is not required for inflammasome function, or that Y36 is phosphorylated in inactivated cells and dephosphorylated upon activation. Therefore, to further elucidate the regulation of ASC by phosphorylation, mimicking of constitutive tyrosine phosphorylation could be carried out to allow the comparison of the two phosphorylation states on ASC functionality and thus enable the determination of whether dephosphorylation or phosphorylation is required for ASC function. However, to introduce a constitutively phosphorylated tyrosine, a synthesized (unnatural) phosphotyrosine would need to be incorporated into the full-length protein at the target site as there is no naturally occurring pTyr mimic. Although glutamate has been used as a pTyr mimic (34), it lacks the chemical and structural similarity to pTyr. This, therefore, means that glutamate substitution introduces a chemical and structural change to the protein of interest, thus not recapitulating the intended constitutive phosphorylation at tyrosine. However, glutamate is better suited as a mimic for phospho-serine and phosphothreonine (35). Therefore, synthetic pTyr mimics are the only option and have so far been incorporated into peptides or by genetic code expansion. However, these two approaches are both highly undeveloped to be utilized in full-length proteins (36). Overall, with the identification of Y60 and Y137 as necessary for inflammasome function, we have shed more light on how the adaptor protein is regulated by tyrosine phosphorylation in inflammasome complex formation.

In conclusion, we have shown that PAO-mediated PTPase inhibition results in the inhibition of the NLRP3 inflammasome via, in part, inhibition of ASC tyrosine dephosphorylation. Our study provides the basis on which to identify the phosphatase(s) involved in ASC regulation and thus have a therapeutic target to treat the symptoms of the various inflammasome-related diseases. We have also identified Y60 and Y137 as putative tyrosine phosphorylation residues adding to the understanding of ASC phospho-regulation in inflammasome complex formation and function.

## Author Contributions

BM, RM, and AM: study concept, design, and writing of the manuscript. BM, KN, HJ, KL, MC, and MV: acquisition, analysis, and interpretation of data. RM and AM: study supervision and fund acquisition.

### Conflict of Interest Statement

The authors declare that the research was conducted in the absence of any commercial or financial relationships that could be construed as a potential conflict of interest.
