# Supplementary material for: Tyrosine Dephosphorylation of ASC Modulates the Activation of the NLRP3 and AIM2 Inflammasomes
Source: Front Immunol. 2019 Jul 5;10:1556. doi: 10.3389/fimmu.2019.01556 (PMC6624653; doi:10.3389/fimmu.2019.01556)
Supplement: Supplementary file 1 [file Data_Sheet_1.PDF]

SUPPLEMENTARY INFORMATION

Tyrosine dephosphorylation of ASC modulates the activation of the NLRP3 and AIM2 inflammasomes

Bezaleel Mambwe, Kurt Neo, Hanif Javanmard Khameneh, Keith Weng Kit Leong, Mariasilvia Colantuoni, Maurizio Vacca, Richmond Muimo, and Alessandra Mortellaro

SUPPLEMENTARY FIGURE

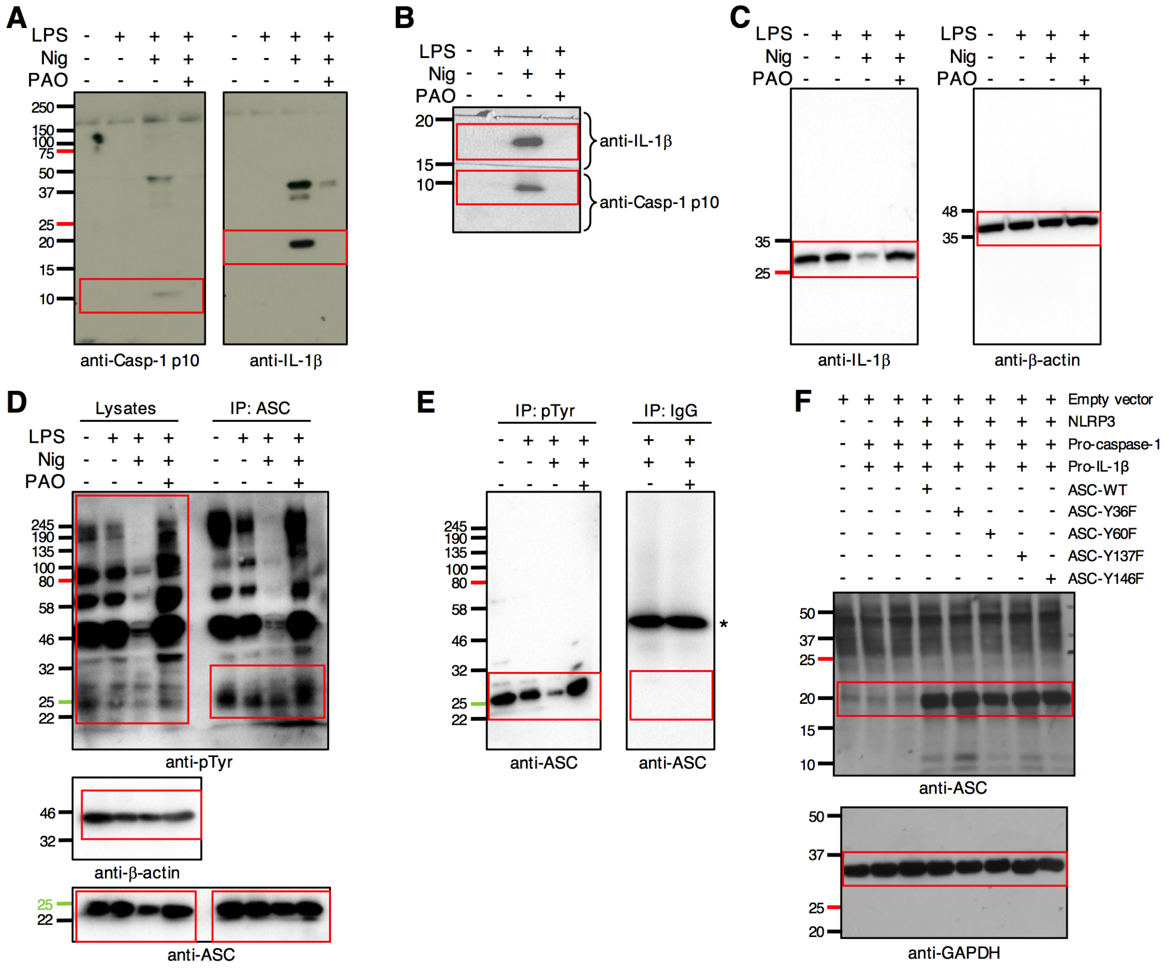

Supplementary Fig. 1. Uncropped blots related to the western blots shown in Figures 1 (A-C), 4 (D, E), and 5 (F). The red boxes highlight the regions of the western blots shown in the figures. * in panel E denotes a non-specific band.
